# Strengthening resilience in Philippine health emergency and disaster risk management: a whole-of-society approach

**DOI:** 10.5365/wpsar.2025.16.5.1208

**Published:** 2025-12-05

**Authors:** Erin L Downey, Ronald Law, Princess Mhyco Esguerra, Rowena Capistrano, Yui Sekitani

**Affiliations:** a World Health Organization, Geneva, Switzerland & Asian Institute of Management, Manila, Philippines.; b Ministry of Health, Manila, Philippines.; c World Health Organization Representative Office for the Philippines, Manila, Philippines.

Strengthening resilience in Health Emergency and Disaster Risk Management (Health EDRM) is a global priority amid rising climate-related and health crises. Whole-of-government (WoG) and whole-of-society (WoS) approaches help countries build adaptive systems that respond to emergencies and mitigate risks from climate change and disasters through cooperation and information-sharing. This case study examines how the Philippines operationalized WoG and WoS strategies at national and local levels, offering lessons for regional and intercountry disaster risk management capacity-building and exchange.

Effective Health EDRM requires a WoG and WoS approach across all pillars, from prevention and mitigation to recovery and rehabilitation. (*1*) These strategies mobilize the government, private sector, civil society, nongovernmental organizations, academia, communities and individuals to collaboratively manage health risks. Inclusive participation harmonizes diverse perspectives and resources, enabling more adaptive and equitable responses to complex, interrelated challenges.

As in other countries in the Western Pacific, the Philippines faces growing climate-related crises, such as extreme weather and shifting ecosystems, which strain Health EDRM. These result in repeated damage to health infrastructure, disruption to supply chains, the exacerbation of noncommunicable conditions, an increased risk of infectious disease transmission due to food and water insecurity, and weakened public health service delivery that cause immediate and long-term consequences for population health. These challenges underscore the urgent need for multifaceted solutions.

In 2013, Typhoon Haiyan (Yolanda), one of the strongest recorded cyclones in the Philippines, caused extensive loss of life, displacement and infrastructure damage, triggering waterborne disease outbreaks from compromised water and sanitation systems. (*2*) Furthermore, the 2016 Zika outbreak highlighted the need for climate-sensitive surveillance and coordinated inter-agency action to manage emerging infectious diseases. (*3*) These complex challenges reinforced efforts by the Government of the Philippines to create structures that integrate climate adaptation, health and disaster risk management into national policy and planning, resulting in the institutionalization of a WoG strategy.

The enactment of Republic Act No. 10121 in 2010 established a multisectoral, inter-agency platform to coordinate disaster risk management and ensure a unified national strategy. (*4*) The National Disaster Risk Reduction and Management Council (NDRRMC) leads and coordinates prevention, preparedness, response and recovery efforts through a multisectoral cluster approach. Within this framework, the Department of Health (DOH) manages the national health system, integrating public health, clinical care, water, sanitation and hygiene, mental health, nutrition and health education under the National Policy on Disaster Risk Reduction and Management in Health (DRRM-H). (*5*)

This inter-agency platform further supports coordinated WoS and WoG efforts by enabling the DOH to collaborate with the Department of Social Welfare and Development to strengthen community resilience through social welfare programmes and equitable access to essential services. Alongside this, the Philippine Health Insurance Corporation also ensures access to quality health care without financial hardship, supports universal health coverage, and covers treatments for disaster-related injuries and illnesses. Additionally, the Department of the Interior and Local Government plays a key role in local disaster response by engaging with local government units, promoting citizen participation, professionalizing local civil service, and strengthening preparedness through coordination, training and resource mobilization.

The DRRM-H policy complements the overall National Disaster Risk Reduction and Management Plan, providing a framework within the health sector by institutionalizing strategies across all governance levels, supporting national and international goals, and clarifying stakeholder responsibilities. Its guiding principles are people-centred, all-hazards, proactive, community-empowered, resilient and evidence-based, ensuring equitable service delivery. The policy is instrumental for protracted crises, such as during COVID-19, when it guided the WoG mechanism between DOH and other NDRRMC agency members.

The country’s COVID-19 response demonstrated the WoS approach through partnerships across multiple sectors outside of government. For example, the Taskforce T3 (Test, Trace, Treat), a private sector-led initiative, was formed in April 2020 to support the national government in its efforts to combat COVID-19. Taskforce T3 played a crucial role in enhancing testing capacity, donating personal protective equipment, and using private sector assets to support the rollout and deployment of vaccines. (*6*)

Another demonstration of WoS was in December 2021, when Typhoon Odette (Rai) struck the Philippines amid the ongoing COVID-19 pandemic. Already burdened by mobility restrictions, communities faced further hardship as the typhoon devastated infrastructure and livelihoods. In response, local governments maintained essential services, for example, by creating a makeshift coordination centre and triage for front-line services, repurposing halls as community kitchens, while community health workers adjusted rotations to support pandemic duties and community needs. (*7*) These efforts were grounded in WoG and WoS approaches, enabling the coordination and mobilization of resources across multiple stakeholders. By promoting shared ownership of preparedness and response, these approaches improve resource efficiency, strengthen coordination from local to national levels, and foster resilience that is responsive to acute crises and long-term systemic risks.

Given the country’s heightened vulnerability to climate-related and other complex emergencies, DOH furthered its commitment to Health EDRM by creating the Health and Climate Change Office in 2025 and underscored its global commitment to the Paris Agreement. National frameworks, such as the National Disaster Risk Reduction and Management Plan 2020–2030 (*8*) and the National Climate Change Action Plan 2011–2028, (*9*) advance the WoG and WoS approach by aligning disaster preparedness with climate resilience and human security imperatives. Recently, the Republic Act No. 12287 institutionalized the declaration of a “State of Imminent Disaster,” which enables the government to provide more preparedness activities and anticipatory actions. (*10*)

Although the Government of the Philippines has made significant strides in advancing Health EDRM through WoG and WoS approaches, several context-specific actions are recommended to further institutionalize these efforts and empower local and regional governments. First, the continued expansion of multisectoral partnerships with the private sector, academia and community organizations would enhance long-term resilience and emergency response. Effective community engagement could be the holding of regular drills and the creation of hazard maps that integrate academic expertise with community knowledge. Second, given the decentralization of responsibilities and resources in the Philippines, it is essential for Health EDRM that the capacity of regional and local governments be strengthened through training and resources. Empowered local authorities are better positioned to deliver faster, more flexible and context-specific responses to emergencies. Third, as climate impacts exacerbate risks to health systems, climate adaptation should be integrated into Health EDRM and be given more concrete actions. This could be achieved by ensuring that the technical expertise and resources are in place to operationalize the existing plans. Lastly, the improvement of health information systems would strengthen surveillance, early warning and preparedness, and enable anticipatory actions, such as pre-positioning supplies, mobilizing health personnel and activating contingency protocols before a hazard becomes a disaster.

The Philippine experience offers transferable lessons for other countries seeking to operationalize WoG and WoS approaches, particularly in linking disaster risk governance, health-system resilience and community-based action in the face of climate-related threats. These lessons highlight the importance of enabling local governments to take the lead in managing health emergencies and disasters, maintaining flexible coordination platforms, and fostering public–private partnerships to manage complex emergencies. It also shows how WoG and WoS approaches enable integrated responses by breaking down silos, particularly where health systems support disaster response and disaster management agencies reinforce health systems during pandemics.
